# Illegitimate Parents

**Published:** 1920-05-15

**Authors:** 


### THE PUBLIC WELL-BEING.

### ILLEGITIMATE PARENTS.

### Some Points in the Commons Debate.

Mr. Neville Chamberlain's Bill, the object of which—broadly stated—is to improve the status of the illegitimate child and divide more equally the responsibility of the parents, was passed at its second reading in the House of Commons by a large majority. But it is doubtful whether it will go much further, because strong opposition to its terms came from the Home Secretary, who intimated that the Government were unable to provide the necessary time for its consideration in detail by the House. With the broad objects of the Bill all speakers were in sympathy, but there was by no means agreement that the detailed provisions of the Bill could be satisfactorily worked. One is inclined, however, to the view that opponents of some of the provisions are over-cautious, especially on the much-disputed clause which compels the mother to disclose the father's name in order that his financial support may be obtained. It is argued that this widens the possibilities of blackmail.

Mrs. Fisher has pointed out that under the existing conditions, the mother can obtain relief by obtaining an affiliation order; but the number of such orders is extremely small in proportion to the number of illegitimate births. The very mother who most needs help, whose child is best worth preserving, is least likely to avail herself of this method. The Bill lays upon the mother the duty of stating, when she registers the child's birth, the name of its father. He will then receive a form from the collecting officer asking if he acknowledges paternity, and, if he does, what financial provision he is prepared to make for his child. If he denies paternity, he is in the position he would occupy under the existing law if an order against him has been applied for; and the mother will have to prove her case to the satisfaction of the magistrates. This should go far to safeguard an innocent man against blackmail, and the new proposals do not give to the blackmailer any more opportunity than she has at present. It is felt by some authorities, however, that it is injudicious to pass an enactment providing that a Government official shall act as prosecutor of a possibly innocent man at the instance of a perjured blackmailer. The comparatively respectable girl, whose need of help is greatest, will not use the affiliation order machinery; but if she is legally obliged to state the name of her child's father she will be guarded against the unscrupulous who endeavour to compel or persuade her to silence, and she will have some chance of caring for her child.

As things are now, it is useless to deny that the mother bears far more than her just share of the burden of irresponsible parenthood, and this frequently reacts to the disadvantage of the unhappy child, who is not properly cared for and is often too eagerly farmed out, the only relationship with its natural protector—the mother—being the burden of weekly payments in support. The registration of the father's name should be a healthy deterrent. Hitherto, in the majority of cases, it is only the mother who suffers the shame of publicity, and if both the father and the mother had the same possibility to look forward to, it might inculcate some sense of responsibility in the man. There would be no necessity to "pillory the parents" if the registration regulations made it possible for the father admitting paternity to come to a reasonable financial arrangement; no proceedings would be required, and it might be done quietly. There is, of course, to be considered the case of the mother who, if the father declined paternity, would shrink from the publicity of court proceedings. But in that event it is the consideration of the welfare of the child which should be paramount, and the mother's capacity to maintain it unaided might be tested before proceedings were instituted. It is not the child that is illegitimate, but the parents, and sentiment ought to be made to give way to the interests of the child. Under the present system, the mother and the child bear nearly the whole of the consequences. Why should they? The subject is serious and urgent enough for the Government to provide what they consider a better alternative to effect the objects of the measure for which the House voted a majority.

